# Barriers and facilitators to the integration of mental health services into primary health care: a systematic review protocol

**DOI:** 10.1186/s13643-017-0561-0

**Published:** 2017-08-25

**Authors:** Edith K. Wakida, Dickens Akena, Elialilia S. Okello, Alison Kinengyere, Ronald Kamoga, Arnold Mindra, Celestino Obua, Zohray M. Talib

**Affiliations:** 1 Office of Research Administration, Mbarara University of Science and Technology, Mbarara, Uganda; 2 Department of Psychiatry, College of Health Sciences, Makerere University, Kampala, Uganda; 3 Africa Center for Systematic Reviews and Knowledge Translation, College of Health Sciences, Makerere University, Kampala, Uganda; 4 Library, Africa Center for Systematic Reviews and Knowledge Translation, College of Health Sciences, Makerere University, Kampala, Uganda; 5 Department of Anatomy, Mbarara University of Science and Technology, Mbarara, Uganda; 6 Department of Pharmacology and Therapeutics and Vice Chancellor, Mbarara University of Science and Technology, Mbarara, Uganda; 7 Department of Medicine & of Health Policy, George Washington University, Washington DC, USA; 8 Mbarara University of Science and Technology, Mbarara, Uganda

**Keywords:** Integration, Mental health services, Primary health care, Barriers and facilitators

## Abstract

**Background:**

Mental health is an integral part of health and well-being and yet health systems have not adequately responded to the burden of mental disorders. Integrating mental health services into primary health care (PHC) is the most viable way of closing the treatment gap and ensuring that people get the mental health care they need. PHC was formally adapted by the World Health Organization (WHO), and they have since invested enormous amounts of resources across the globe to ensure that integration of mental health services into PHC works.

**Methods:**

This review will use the SPIDER (Sample, Phenomenon of Interest, Design, Evaluation, Research type) framework approach to identify experiences of mental health integration into PHC; the findings will be reported using the “Best fit” framework synthesis. PubMed, EMBASE, PsycINFO, and Cochrane Central Register of Controlled trials (CENTRAL) will be searched including other sources like the WHO website and OpenGrey database. Assessment of bias and quality will be done at study level using two separate tools to check for the quality of evidence presented.

Data synthesis will take on two synergistic approaches (qualitative and quantitative studies). Synthesizing evidence from countries across the globe will provide useful insights into the experiences of integrating mental health services into PHC and how the barriers and challenges have been handled. The findings will be useful to a wide array of stakeholders involved in the implementation of the mental health integration into PHC.

**Discussion:**

The SPIDER framework has been chosen for this review because of its suitable application to qualitative and mixed methods research and will be used as a guide when selecting articles for inclusion. Data extracted will be synthesized using the “Best fit” framework because it has been used before and proved its suitability in producing new conceptual models for explaining decision-making and possible behaviors. Synthesizing evidence from countries across the globe will provide useful insights into the experiences of integrating mental health services into PHC and how the barriers and challenges have been handled.

**Systematic review registration:**

PROSPERO CRD42016052000

**Electronic supplementary material:**

The online version of this article (doi:10.1186/s13643-017-0561-0) contains supplementary material, which is available to authorized users.

## Background

### Description of the condition

Mental health as defined by the World Health Organization is a state of well-being in which every individual realizes his or her own potential, can cope with the normal stresses of life, can work productively and fruitfully, and is able to make a contribution to her or his community [1]. It is an integral part of health and well-being and yet health systems are yet to adequately respond to the burden of mental health problems. Up to 85% of people with severe mental disorders in low-income and middle-income countries receive no treatment for their disorder [2, 3]. Mental and behavioral disorders are estimated to account for 14% of the global burden of disease with 19% of the burden in sub-Saharan Africa (SSA) [4]. If untreated, mental and behavioral disorders are likely to cause severe disability and heavy socio-economic burden on families and communities [5–9]. Integrating mental healthcare services into primary health care (PHC) is among the most viable means of closing the treatment gap and ensuring that people get the mental healthcare they need [7, 9].

### Description of the intervention

PHC is the first point of contact an individual has with the health system and is essential to making health care universally accessible to individuals and families in the community in an acceptable and affordable way with full participation [10, 11]. It was formally adopted by the WHO through the Alma-Ata declaration as the best method for providing a comprehensive, universal, equitable and affordable healthcare service and that it had the ability to reduce stigma, improve access to care, reduce chronicity of mental illness and improve social integration [4, 12, 13]. The Alma-Ata model of mental health integration recommends that countries build or transform their mental health services to (i) promote self-care, (ii) build informal community care services, (iii) build community mental health services, (iv) develop mental health services in general hospitals, and (v) limit reliance on psychiatric hospitals [14].

Furthermore, evidence suggests that mental health care can be delivered effectively in PHC settings, and that once identified, most mental illnesses can be treated using cost-effective means [9, 15, 16]. Treatment of common mental disorders at PHC can be improved through collaborative care interventions, yielding better access to care, better physical as well as mental health outcomes, and improved overall cost-effectiveness [17, 18].

### How the intervention might work

The WHO issued key recommendations to guide the process of integrating mental health services into PHC; these include (i) doing a preliminary situational analysis of the best options for the treatment and care of mental disorders at different levels of care, (ii) building on existing networks/structures and human resources to provide mental health services, (iii) redistributing funding from tertiary to secondary and primary levels of care or making new funds available, (iv) clear delineation of mental disorders to be treated at the primary care level, (v) training of primary care staff in identification and treatment of mental disorders (vi) recruitment/education of new PHC staff, (vii) availing of basic psychotropic medicines at primary and secondary care levels, and (viii) adequate supervision and support of PHC staff by mental health specialists if integration is to succeed [4].

### Why it is important to do this review

There is a need to strengthen health systems to address the existing treatment gaps for mental health given that the WHO has invested enormous amounts of resources to ensure that integration of mental healthcare services into PHC is achieved [19].

Furthermore, health systems have both the hardware (human resources, finance, medicines and technology, service infrastructure, and information system) as well as the software (ideas and interests, relationships and power, values and norms) [20] at whose center are people. Without putting people first, there are bound to be challenges of integrated healthcare because no established direct relationship between individuals, families in the community, and a specific staff member have been created [7].

Integration of mental health into PHC has been done by various countries and in different forms [21–24] including training PHC workers to identify mental health problems, assessing for mental illnesses during medical standard of care, PHC providers/community health workers and health care managers working together to address mental health related illnesses, and availing psychotropic medications to PHC centers [25]. In addition, researchers have documented information about barriers and facilitators to the integration process [26–28]. Integration of mental health services, for the purpose of this review, will be defined as blending mental healthcare services (identification, treatment, and or referral) into the medical standard of care. PHC will be defined as the first point of contact with the health system [29].

The review will be based on the SURE (Supporting the Use of Research Evidence) guide that focuses on barriers to implementing health system changes [30, 31] (see Table 1). The SURE framework is among the most robust with regards to the identification of and addressing barriers to implementing policy options; however, it was developed for implementing health changes within Africa. This review will in part validate the SURE guide for global use. It will synthesize and document the barriers and facilitators to the integration of mental health services into PHC and explain the implementation of mental health integration into PHC. The results will be useful to key stakeholders involved in the implementation of the mental health integration into PHC.

**Table 1:** SURE framework for identifying factors affecting implementation of a policy

Level	Factors affecting implementation
Recipients of care	Knowledge and skills
	Attitudes regarding program acceptability, appropriateness and credibility
	Motivation to change or adopt new behavior
Providers of care	Knowledge and skills
	Attitudes regarding program acceptability, appropriateness and credibility
	Motivation to change or adopt new behavior
Other stakeholders (including other healthcare providers, community health committees, community leaders, program managers, donors, policy makers and opinion leaders)	Knowledge and skills
	Attitudes regarding program acceptability, appropriateness and credibility
	Motivation to change or adopt new behavior
Health system constraints	Accessibility of care
	Financial resources
	Human resources
	Educational system
	Clinical supervision
	Internal communication
	External communication
	Allocation of authority
	Accountability
	Management and or leadership
	Information systems
	Facilities
	Patient flow processes
	Procurement and distribution systems
	Incentives
	Bureaucracy
	Relationship with norms and standards
Social and political constraints	Ideology
	Short-term thinking
	Contracts
	Legislation or regulations
	Donor policies
	Influential people
	Corruption
	Political stability

Adopted from *“The SURE Collaboration, 2011” World Health Organization*

## Review objectives

The objectives of this review are to synthesize and document the barriers and facilitators to the integration of mental healthcare services into primary health care.

## Methods/design

The systematic review is registered in PROSPERO international prospective register of systematic reviews (CRD42016052000). It has been written according to the Preferred Reporting Items for Systematic Reviews and Meta-Analyses Protocols (PRISMA-P) recommended for systematic reviews [32]. The checklist is included as an additional file (see Additional file 1). This review will use the SPIDER (Sample, Phenomenon of Interest, Design, Evaluation, Research type) framework approach [33] and the findings will be reported using the “Best fit” framework synthesis [34].

### Sample

The sample or population of interest is health care providers, community health workers, health care managers, and policy makers who have been involved in the integration of mental health into PHC. The listed stakeholders should be involved in general health care, collaborative care, and/or specialized health care. We will include studies from any country in the world involved in the integration of mental health services into PHC.

### Phenomenon of interest

We will include studies that document the integration of mental health services into general healthcare; are delivered at primary or community health care settings; and are collaborative in nature (the primary health care providers, community health workers, and health care managers, working together) so as to understand the how and why of certain behaviors, decisions, and individual experiences of integration of mental health into PHC.

### Design

The theoretical framework in the studies will be used to determine the research method used, while the details of the study design will help to make decisions about the robustness of the study and analysis. In addition, this might increase the detection of qualitative studies in the databases in which titles and abstracts are unstructured [33].

### Evaluation

Evaluation of the outcomes will be done depending on the research question which may include unobservable and subjective constructs like attitudes and views when compared to quantitative research [33].The outcomes will be looked at in relation to the SURE Framework as detailed in Table 1 to evaluate the policy option.

### Research type

We will review studies that have documented experiences and attitudes of stakeholders about integration of mental health into general PHC; studies conducted in specialized clinics (HIV, maternal and child health, and reproductive health) will be included. Our search will cover three research types: (i) qualitative studies that used appropriate methods of data collection and analysis (such as ethnography, grounded theory, phenomenology, and case studies) [31, 35, 36]; (ii) quantitative studies; and (iii) mixed methods studies combing qualitative and quantitative methods of data collection and analysis which will include cross-sectional studies, case-control studies, cohort studies, quasi-experimental studies, and randomized control trials.

### Inclusion criteria

To be eligible for inclusion, the articles will be required to describe mental health integration (policy and, or service provision) in PHC settings. They should involve one or more type of care provider (doctor, nurse, social worker, clinical officer, community health worker); deal with any type of mental health (depression, schizophrenia, anxiety, or general mental health) and any age group or population that receive mental health services; and present barriers/challenges, facilitators/enablers of mental health integration into PHC. Articles with aspects of collaborative engagements (primary care providers with mental health specialists and other professionals) will be eligible for inclusion. See Table 2 for categorization of inclusion criteria by SPIDER framework.

**Table 2:** Inclusion criteria

	Primary research studies
Setting/population	Primary health care/community
Phenomenon of interest	Mental health integration (policy, and/or service provision)
Design, evaluation, and research	Interviews, focus group discussions, surveys (that explored primary care providers’ experiences (barriers and challenges/facilitators and enablers), perceptions, attitudes

Articles not in a PHC or community setting, offering training in mental health integration, and conducting a situation analysis for integration of mental health into PHC will not be eligible for inclusion. The quality of reporting of studies will be considered when selecting articles for inclusion, and inadequately reported studies will be excluded [37].

## Search methods for identification of studies

We will search the following databases to identify potentially eligible studies for review: PubMed, EMBASE, PsycINFO, and Cochrane Central Register of Controlled trials (CENTRAL). The search will be open to any language, no date restriction will be placed, and the search terms will be kept broad to capture potentially eligible studies. An example of the proposed search strategy of PubMed is attached as Additional file 2. In addition, other sources including the WHO website, OpenGrey database, and reference lists from relevant studies reviewed will be searched. Where necessary, experts or specialist authors in the field will be contacted.

## Data extraction

Two review authors (EW and RK) will independently screen the titles and/or abstracts of the identified records for eligibility. The full text of all the papers identified as potentially relevant will then be retrieved by AK and DA. Eligible citations will be read in full-text version by EW, EO, and ZT and evaluated for inclusion using the eligibility criteria. Disagreements will be resolved by consensus and discussion with a third reviewer (CO) when needed. Following the literature search, an EndNote database (EndNote version X7.7.1 Thomson Reuteurs) will be used to manage search results.

Three review authors (EW, AM, and RK) will independently extract data using a pre-tested data extraction form; discrepancies will be identified and resolved through discussion (with the senior researcher on the team CO where necessary). Where appropriate, we (EW) will contact the corresponding authors of the included studies for clarification and missing data.

Extracted data will include study title, name of the first author, year of publication, country of study, study setting, facility type (public/free at point of use, insurance based, private/nominal payment, NGO, etc.), study type (qualitative, quantitative and mixed methods studies), study population/cadre (health care providers/community health workers,/health care managers including doctors, nurses, clinical officers, social workers among other health cadres and policy makers), and barriers and facilitators (categorized by study type).

The key information will be identified through full-text review and extracted by study type (qualitative/quantitative barriers and facilitators separately), after which it will be categorized along the parameters in the SURE framework [30] as detailed in Table 1 in preparation for synthesis.

This review derives its elements from three sources: (1) key information required (population, setting, and intervention for synthesis and interpretation, (2) SURE framework to guide the implementation of health systems changes, and (3) the quality assessment criteria. Two reviewers will conduct independent quality assessments of the included studies [37] so as to inform judgment of not only the internal validity of included studies but also the validity of the findings of the synthesis [34].

## Risk of bias and quality assessment

Assessment of bias and quality will be done at study level using two separate tools to check for the evidence presented. For qualitative studies, the Critical Appraisal Skills Program (CASP) qualitative checklist [38] will be used while the Effective Public Health Practice Project Quality Assessment Tool will be used for Quantitative Studies [39]. In order to limit publication bias, articles from both published and unpublished data sources will be included, information will be compared to ensure that it is representative of completed studies conducted in the same population, studies published in any language will be included, and there will be no selective reporting of some outcomes to the exclusion of others. This process will be ensured by the primary reviewer (EW) in consultation with EO and ZT.

## Primary outcomes

The primary outcomes of the review will be the barriers (challenges) and facilitators (enablers) to the integration of mental health services into primary health care. We have chosen to use the terms “barriers and facilitators” to describe all factors that might inhibit or facilitate the implementation of a policy option [30] which in this case is the integration of mental health into PHC. Barriers will be defined as all factors that create obstacles or prevent progress towards attaining set goals whilst facilitators are all factors that make it easy or easier to attain a set goal or complete a set task. These “factors” may be physical or metaphorical. The content of the barriers and facilitators will be defined by the SURE guide. The “findings unit” shall either be predefined by the author or identified in a verbatim quote. In order to synthesize results across the included studies, a review of the reported outcomes for each study will be done [40] by EW and ZT to determine how the primary outcome of interest has been categorized along the SURE framework and reported for each study.

## Data synthesis

Data synthesis will take on two synergistic approaches given that we plan to include both qualitative and quantitative studies. In the process of data extraction, key information will be categorized by study type and given codes which will be used in coding framework. The synthesis will adapt the “Best fit” framework synthesis by making reference to the extracted data from the included studies to construct a new evidence-based conceptual model regarding integration of mental health into primary health care [34]. The conceptual framework will be composed of a priori themes supported by evidence from the studies, plus new themes generated by the thematic analysis of evidence falling outside the framework. The conceptual framework will be assessed for bias and to determine if the synthesis is sensitive to the adjudged reported quality, design or location of included studies. Any differences between the a priori framework and the new framework will be explored when the synthesis is complete. This will test publication bias within the sample of included studies as well as the SURE framework for global use.

The process will be overseen by EO, a qualitative research methods expert on the review team, to ensure reflexivity, rigor, and quality.

## Discussion

The SPIDER framework has been chosen for this review because of its suitable application to qualitative and mixed methods research and will be used as a guide when selecting articles for inclusion [33, 34]. We intend to leave our search open to any language with no date restriction and with broad search terms to capture potentially eligible studies that will ensure that our research question is answered. Data will be independently extracted to promote reflexivity during the process. Quality assessment tools will be applied to the selected articles to ensure quality of evidence presented.

Data extracted will be synthesized using the “Best fit” framework because it has been used before and proved its suitability in producing new conceptual models for explaining decision-making and possible behaviors [34]. In addition, evaluation of the review findings which will include attitudes and views among other unobservable and subjective constructs will enable us to understand the how and why of certain behaviors, decisions, and individual experience towards integration of mental health services into PHC. Synthesizing evidence from countries across the globe will provide useful insights into the experiences of integrating mental health services into PHC and how the barriers and challenges have been handled.

The findings will be used to explain reasons affecting implementation, predict how stakeholders respond, and/or identify areas that are not functioning well within the health system [41]. They will be useful to a wide array of stakeholders involved in the implementation of the mental health integration into PHC. Examples of unsuccessful integration of mental health will not cause publication bias but simply present global experiences and best practices.

## Additional files

Additional file 1: PRISMA-P. (DOCX 31 kb)

Additional file 2: Search string. (DOCX 15 kb)

## Abbreviations

CASP: Critical Appraisal Skills Program
PHC: Primary health care
SURE: Supporting the Use of Research Evidence
WHO: World Health Organization
