# Supplementary material for: Barriers and facilitators to the integration of mental health services into primary health care: a systematic review protocol
Source: Syst Rev. 2017 Aug 25;6:171. doi: 10.1186/s13643-017-0561-0 (PMC6389192; doi:10.1186/s13643-017-0561-0)
Supplement: Supplementary file 2 — Search string. (DOCX 15 kb) [file 13643_2017_561_MOESM2_ESM.docx]

**Additional file 2: Search Strategy in PubMed**

| Concept | Description of concept | Research Terms |
| --- | --- | --- |
| A | Mental Health | “Mental health”[Mesh] OR “Mental health[Text Word] OR Mental health[tiab] OR Mental health service*[Mesh] OR Mental health services*[tiab] OR Health services, mental[tiab] OR Health service, mental[Mesh] OR Service, mental health[Mesh] OR mental illness*[Mesh] OR mental disease*[Text Word] OR psychiatric condition*[Text Word] OR psychiatric diseases*[Text Word] OR psychiatric disorder*[Text Word] OR mental health service*[Text Word] OR mental health care[Text Word]))) |
| B | Integrate | Integrate OR integration[Text Word] |
| C | Primary Health Care | Primary Health care OR Primary Healthcare OR Community health care OR Community healthcare OR PHC OR Primary Care |

Search strategy « A » & « B » & « C »
